# Aging Impairs Hippocampal- Dependent Recognition Memory and LTP and Prevents the Associated RyR Up-regulation

**DOI:** 10.3389/fnagi.2017.00111

**Published:** 2017-04-25

**Authors:** Alejandra Arias-Cavieres, Tatiana Adasme, Gina Sánchez, Pablo Muñoz, Cecilia Hidalgo

**Affiliations:** ^1^Biomedical Neuroscience Institute, Faculty of Medicine, Universidad de ChileSantiago, Chile; ^2^Centro Integrativo de Biología y Química Aplicada, Universidad Bernardo O’HigginsSantiago, Chile; ^3^Pathophysiology Program, Institute of Biomedical Sciences, Faculty of Medicine, Universidad de ChileSantiago, Chile; ^4^Center for Applied Neurological Sciences and Interdisciplinary Center for Innovation in Health, School of Medicine, Universidad de ValparaísoValparaíso, Chile; ^5^Center of Molecular Studies of the Cell and Physiology and Biophysics Program, Institute of Biomedical Sciences, Faculty of Medicine, Universidad de ChileSantiago, Chile

**Keywords:** calcium release, synaptic plasticity, RyR oxidation, behavior, synaptic plasticity, gene expression

## Abstract

Recognition memory comprises recollection judgment and familiarity, two different processes that engage the hippocampus and the perirhinal cortex, respectively. Previous studies have shown that aged rodents display defective recognition memory and alterations in hippocampal synaptic plasticity. We report here that young rats efficiently performed at short-term (5 min) and long-term (24 h) hippocampus-associated object-location tasks and perirhinal cortex-related novel-object recognition tasks. In contrast, aged rats successfully performed the object-location and the novel-object recognition tasks only at short-term. In addition, aged rats displayed defective long-term potentiation (LTP) and enhanced long-term depression (LTD). Successful long-term performance of object-location but not of novel-object recognition tasks increased the protein levels of ryanodine receptor types-2/3 (RyR2/RyR3) and of IP_3_R1 in young rat hippocampus. Likewise, sustained LTP induction (1 h) significantly increased RyR2, RyR3 and IP_3_R1 protein levels in hippocampal slices from young rats. In contrast, LTD induction (1 h) did not modify the levels of these three proteins. Naïve (untrained) aged rats displayed higher RyR2/RyR3 hippocampal protein levels but similar IP_3_R1 protein content relative to young rats; these levels did not change following exposure to either memory recognition task or after LTP or LTD induction. The perirhinal cortex from young or aged rats did not display changes in the protein contents of RyR2, RyR3, and IP_3_R1 after exposure at long-term (24 h) to the object-location or the novel-object recognition tasks. Naïve aged rats displayed higher RyR2 channel oxidation levels in the hippocampus compared to naïve young rats. The RyR2/RyR3 up-regulation and the increased RyR2 oxidation levels exhibited by aged rat hippocampus are likely to generate anomalous calcium signals, which may contribute to the well-known impairments in hippocampal LTP and spatial memory that take place during aging.

## Introduction

In diverse animal species, the aging process usually entails synaptic transmission and plasticity deficits that occur in different brain regions and correlate with learning and memory impairments (Foster and Norris, 1997; Barnes, 2003; Disterhoft et al., 2004). In particular, aging impairs recognition memory, defined as the ability to remember a previously presented item (Warburton and Brown, 2010) and which entails familiarity and recollection judgment. Familiarity depends on the function of the PrhC and involves recognition of previously presented items (Haskins et al., 2008). Recollection judgment relies on hippocampal function and relates to context or spatial location (Warburton and Brown, 2010).

Neuronal Ca^2+^ signals have key roles in modulating LTP, LTD (Mulkey and Malenka, 1992) and activity-dependent gene expression (Bading, 2013). At the cellular level, aging commonly results in defective Ca^2+^ signaling (Oliveira et al., 2014) and oxidative stress, caused by increased production of ROS that overcomes the cellular antioxidant systems (Sohal and Orr, 2012). In turn, oxidative stress alters Ca^2+^ signaling by promoting redox modifications of key proteins engaged in Ca^2+^ homeostasis and signaling (Paula-Lima et al., 2014; Hidalgo and Arias-Cavieres, 2016). Given that anomalous Ca^2+^ signaling during aging leads to significant perturbations of neuronal function (Gant et al., 2006, 2015), studying the cellular mechanisms underlying impaired Ca^2+^-signaling during aging is important to decipher, at least partly, the synaptic transmission deficits and cognitive decline associated to aging.

A number of Ca^2+^-dependent electrophysiological processes undergo age-dependent changes, (Disterhoft et al., 1996; Foster and Norris, 1997; Thibault et al., 2001; Gant et al., 2006, 2014, 2015; Bodhinathan et al., 2010b). These changes are consistent biomarkers of aging and correlate with cognitive decline (Disterhoft et al., 1996; Gant et al., 2006; Disterhoft and Oh, 2007). In addition, age-related increases in Ca^2+^ influx through L-type voltage-gated Ca^+2^ channels are associated with hippocampal electrophysiological and cognitive defects (Thibault et al., 2001; Veng et al., 2003; Disterhoft et al., 2004). Neuronal Ca^2+^ signals also arise from Ca^+2^ release mediated by ryanodine receptor (RyR) and inositol 1,4,5-triphosphate receptor (IP_3_R) channels present in the endoplasmic reticulum of dendrites and axons (Verkhratsky, 2005). In young rodents, Ca^2+^ signals produced by activation of Ca^2+^ release channels from intracellular stores have key roles on hippocampal-dependent memory and normal synaptic plasticity (Lu and Hawkins, 2002; Raymond, 2007; Galeotti et al., 2008; Adasme et al., 2011; Grigoryan et al., 2012; Baker et al., 2013; Paula-Lima et al., 2014). In aged neurons, aberrant activation of Ca^2+^ release through RyR channels contributes significantly to defective neuronal function (Gant et al., 2006; Paula-Lima et al., 2014).

Changes in neuronal redox state, such as the increased oxidative tone exhibited by aged neurons, affect in particular RyR-mediated Ca^2+^ release (Paula-Lima et al., 2014), since RyR channels are highly redox-sensitive (Hidalgo and Donoso, 2008). Of note, RyR channel inhibition or antioxidant agents significantly decrease the sustained Ca^2+^-dependent sAHP exhibited by aged hippocampal neurons (Bodhinathan et al., 2010b). Hence, it is highly likely that enhanced Ca^2+^ release caused by RyR channel oxidation contributes to this anomalous sAHP response, which by decreasing neuronal excitability contributes to age-related hippocampal dysfunction and memory decline; however, we have not found reports describing RyR channel oxidation levels in aged neurons.

The hippocampus expresses the three RyR mammalian isoforms (Furuichi et al., 1994; Hertle and Yeckel, 2007). Young rats trained in the Morris water maze exhibit increments in the hippocampal protein levels of the RyR2 (Zhao et al., 2000) and the RyR2/RyR3 (Adasme et al., 2011) isoforms, while selective knockdown of the RyR2 or the RyR3, but not of the RyR1 isoform inhibits avoidance memory processes in rodents (Galeotti et al., 2008). Memory processes in young rodents require functional RyR channels, since inhibition of RyR activity with dantrolene reduces associative memory (reviewed in Baker et al., 2013), and intra-hippocampal injection of ryanodine at a concentration that stimulates RyR activity increases spatial memory acquisition and consolidation (Adasme et al., 2011). In contrast, dantrolene attenuates age-associated spatial memory deficits (Hopp et al., 2014). The positive effects of dantrolene on aged animals may be due to inhibition of excessive RyR-mediated Ca^2+^ release, which as discussed below, is likely to have a negative impact on spatial memory processes during aging. Moreover, in aged rats, the hippocampal mRNA levels of all three RyR isoforms display an inverse correlation with performance in the Morris water maze (Hopp et al., 2014).

Of the three mammalian IP_3_R isoforms, IP_3_R1 represents the most abundant IP_3_R isoform present in brain (Hertle and Yeckel, 2007); however, we have not found reports describing activity-related or aged-related changes in IP_3_R1 hippocampal expression. Likewise, no reports are available describing hippocampal RyR protein content in aged animals or RyR or IP_3_R1 protein content in the PrhC. Consequently, in this work we measured in young or aged rats RyR1/RyR2/RyR3 mRNA levels, and RyR2/RyR3 and IP_3_R1 protein content in the hippocampal CA1 region and PrhC, before and 24 h after training in recognition memory tasks, and in the hippocampal CA1 region following LTP and LTD induction. We also evaluated recognition memory, hippocampal LTP and LTD in young and aged rats, and determined RyR2 channel oxidative state in the hippocampus from young or aged rats.

## Materials and Methods

### Animals

Young (3 months old) and aged (18–24 months old) male *Sprague Dawley* rats were obtained from the Universidad de Valparaíso animal facility. Food and water were provided *ad libitum*. Lights were maintained on a 12–12 light/dark cycle and all experiments were performed in the light phase. Animals were handled in the experimental room for 10 min per day for 3 days prior to the first exposure to the behavioral apparatus. All experimental protocols used in this work complied with the “Guiding Principles for Research Involving Animals and Human Beings” of the American Physiological Society and were approved by the Bioethics Committee for Investigation in Animals of the Universidad de Valparaíso, Valparaiso, Chile.

### Recognition Memory Tasks

Both the object-location and the novel-object recognition memory tasks were performed as described, with minor modifications (Uekita and Okanoya, 2011; Wimmer et al., 2012). The behavioral procedure included three consecutive phases: open field exploration, sample phases and recognition memory tasks. During the open field exploration phase, rats were habituated to the arena for 5 min during three consecutive days, using as behavioral apparatus a polyethylene black box (50 cm × 40 cm × 63 cm). During the sample phases, each rat was placed in the apparatus and was allowed to explore four different objects during 5 min for three consecutive sessions, separated by 5 min intervals. Four groups of young and four groups of aged rats were evaluated independently in the object-location or the novel-object recognition memory tasks at short-term (5 min) or long-term (24 h). To test object-location memory, two of the four objects previously presented in the sample phases were repositioned and the times exploring the repositioned and the non-relocated objects, and the quiescent times were determined as detailed below. To test object recognition memory, one familiar object was replaced with a novel-object and the times exploring the novel and the familiar objects, and the quiescent times were determined as described below. To eliminate odor cues between trials, the experimental apparatus and all objects were cleaned with 75% ethanol after each trial. Rat behavior was recorded with a video camera positioned over the behavioral apparatus and the collected videos were analyzed with the ANY-MAZE software (Stoelting Co., Wood Dale, IL, USA).

### Hippocampal Slice Preparation

Six hours after concluding the behavioral procedures, animals under halothane anesthesia were sacrificed by decapitation and their brains were quickly removed. The hippocampal tissue was removed, dissected, immersed in cold dissection buffer (in mM: 212.7 sucrose, 5 KCl, 1.25 NaH_2_PO_4_, 2 MgCl_2_, 1 CaCl_2_, 26 NaHCO_3_ and 10 glucose, pH 7.4) and cut into 400 μm transversal slices with a vibratome (Vibratome 1000 plus, Ted Pella Inc., Redding, CA, USA). Likewise, the perirhinal and surrounding cortex were immersed in cold dissection buffer and cut into 400 μm slices as described (Ziakopoulos et al., 1999). The hippocampal slices used for electrophysiological experiments were transferred to an immersion storage chamber and were kept at room temperature for 1 h in artificial cerebrospinal fluid (ACSF) solution (in mM: 124 NaCl, 5 KCl, 1.25 NaH_2_PO_4_, 1 MgCl_2_, 2 CaCl_2_, 26 NaHCO_3_, 10 glucose, pH 7.4), bubbled with 95% O_2_/ 5% CO_2_. For mRNA or protein determinations (see below), the CA1 region or the PrhC from slices collected from controls or trained rats were micro-dissected and stored at -80°C for subsequent analysis.

### Hippocampal Electrophysiology

Electrophysiological experiments were performed in an immersion-recording chamber. To evaluate fEPSP hippocampal slices were superfused at 30 ± 2°C with artificial cerebro-spinal fluid (ACSF) bubbled with 95% O_2_ / 5% CO_2_, at a rate of 2 ml/min. To evoke fEPSP, square current pulses (0.2 ms) were delivered with a concentric bipolar stimulating electrode (FHC Inc., Bowdoinham, ME, USA) located in the Schaeffer collateral–commissural fibers; fEPSP were recorded with ACSF-filled glass microelectrodes (2–3 MΩ) placed into the *stratum radiatum* layer of the CA1 region. To evaluate basal excitatory synaptic transmission, pulses of 25, 50, 75, 100, 150, or 200 microamperes were applied to generate an input/output curve. Results are presented as stimulus intensity versus FV amplitude or fEPSP slope. To evaluate pre-synaptic response components, two pulses were applied every 15 s, with inter-stimulus intervals starting at 20 ms and ending at 640 ms, doubling the interval after each trial. Results from paired-pulse stimulation experiments are presented as the ratio between the values of the initial fEPSP slope evoked by the second over the first stimulus. After monitoring both basal synaptic transmission and pre-synaptic responses, we evaluated LTP adjusting fEPSP to half of the maximal evoked response. Pulses were applied every 15 s until a stable baseline was recorded for at least 15 min. To induce LTP, we used the TBS protocol, comprised of four trains of 10 bursts at 5 Hz each, where each burst comprised four pulses at 100 Hz. To induce LTD, we used LFS protocols (1 Hz / 900 pulses). In all experiments, fEPSP were recorded for 60 min after applying the TBS or LFS protocols. Recordings were filtered at 10 kHz and digitized at 5 kHz using Igor Pro (WaveMetrics Inc., Lake Oswego, OR, USA). Synaptic responses were quantified as the initial slope of the evoked fEPSPs and were plotted as percentage of basal change, defining as 100% the slope measured during baseline recording.

### RNA Isolation and qRT-PCR

Total RNA was isolated using Trizol reagent (Invitrogen, Carlsbad, CA, USA); DNA digestion with DNA-free^TM^ Kit (Ambion, Austin, TX, USA) was included to remove any contaminating genomic DNA. RNA purity was assessed by the 260/280-absorbance ratio and RNA integrity by gel electrophoresis (Adasme et al., 2011). cDNA was synthesized using 2 μg total RNA with the Improm TM II reverse transcriptase (Promega, Madison, WI, USA). Two hundred nanogram of cDNA in a final volume of 20 μl was used for qPCR amplification, which was performed in the Stratagene MX3000P QPCR System (La Jolla, CA, USA) using the DNA binding dye SYBR green (Brilliant III Ultra-fast SYBR^®^ Green QPCR Master Mix; Carlsbad, CA, USA). For each analyzed gene, 10 picomoles of forward and reverse primers were used. The primer sequences used are listed in **Table 1**. To determine the level of RyR relative to that of β-actin mRNA we used the 2^-ΔΔCT^ method (Pfaffl, 2001).

**Table 1:** Primer sequences used.

	forward	reverse
RyR1^1^	GGTGGCCTTCAACTTCTTCC	ACTTGCTCTTGTGGTCTCG
RyR2^1,2^	CTACTCAGGATGAGGTGCAGA	CTCTCTTCAGATCCAAGCCA
RyR3	GAAGCCTGTTGGACCATA	TCCAGAGTGTTTGCATAAAGGAG
β-actin	TCTACAATGAGCTGCGTGTG	TACATGGCTGGGGTGTTGAA

*All primer sequences correspond to 5′ to 3′. ^1^Adasme et al., 2011. ^2^Zhao et al., 2000.*

### Western Blot Analysis

Micro dissections of the hippocampal CA1 region and the PrhC from young and aged rats were homogenized in lysis buffer A (in mM: 300 sucrose, 2 EDTA, 2 EGTA, 1 BAPTA, 20 MOPS, pH 7.0, 1% Nonidet P-40, 0.1 % SDS) containing protease inhibitors (Calbiochem, La Jolla, CA, USA). Lysates were sonicated five times for 20 s with 20 s intervals, and were centrifuged at 4.000 rpm for 20 min to remove debris. After determining protein concentration with the sulfosalicylic acid Protein Assay Kit (Thermo Scientific, Rockford, IL, USA), the resulting supernatants were diluted with Laemmli buffer and loaded on SDS-containing discontinuous polyacrylamide gradient gels (4.5 and 15%). After electrophoresis, gels were transferred to PVDF membranes (Millipore Corp., Bedford, MA, USA) using Transfer-Blot^®^ Turbo System (BIO-RAD, Hercules, CA, USA); membranes were subsequently blocked for 1 h at room temperature with 5% non-fat milk for β-actin, RyR2 and IP_3_R1 or with 5% bovine serum albumin (BSA) in Tris-buffered saline (TBS), pH 7.4, for RyR3 and washed with TBS-Tween 20% (TBS-T). Membranes were incubated under constant shaking with primary antibodies: mouse anti-RyR2 (1:4000, Thermo, Waltham, CA, USA), rabbit anti-IP_3_R1 (1:4000, Thermo, Waltham, CA, USA), or mouse β-actin (1:20000, Sigma, San Luis, MI, USA). Incubations were performed at 4°C overnight in 5% non-fat milk containing 0.2% Tween-20. For RyR3 immunodetection, membranes were incubated as above with rabbit anti-RyR3 (1:4000, Millipore, Bedford, MA, USA) dissolved in TBS-T containing 5% BSA. After washing three times with TBS-T for 10 min, membranes were incubated for 1.5 h at room temperature with appropriate secondary antibodies. Finally, membranes were washed three times with TBS-T for 10 min and immunoreactive proteins were detected with enhanced chemiluminescence (ECL) reagents according to the manufacturer instructions (Amersham Biosciences, Piscataway, NJ, USA). Signals were captured with the ChemiDoc system (Bio-Rad, Hercules, CA, USA). The IMAGE J image program (National Institutes of Health, USA) was used to quantify optical band intensity.

### Determination of RyR2 Free Sulfhydryl (SH) Content

The whole hippocampus was homogenized in lysis buffer B (0.3 M sucrose, 20 mM Tris-MOPS, pH 7.0, plus protease inhibitors: 20 μg/ml benzamidine, 1 μg/ml leupeptin, 1 μg/ml pepstatin, 10 μg/ml trypsin inhibitor and 20 μg/ml phenylmethylsulfonyl fluoride). The homogenate was sedimented at 4,000 g for 10 min at 4°C. The resulting supernatant was sedimented at 100,000 × *g* for 1 h, the pellet was homogenized in lysis buffer B. After determining protein concentration with the sulfosalicylic acid Protein Assay Kit, the resulting suspension was bubbled with argon gas and stored at -80°C. To label free SH residues, frozen aliquots were thawed and diluted to 2 mg/ml with Tris-HCl buffer, pH 7.4, and were incubated for 1 h on ice with 0.4 nmol/mg protein of EZ-Link Maleimide-PEG2-Biotin (Thermo Scientific, Rockford, IL, USA), followed by further incubation for 30 min with glutathione (2 nmol/mg of protein). After protein separation by SDS-PAGE electrophoresis and transfer to PVDF membranes as above, blots were blocked with 5% non-fat milk in TBS-T and incubated for 1 h at room temperature with streptavidin reagent (1:50,000, Thermo Scientific, Rockford, IL, USA). Membranes were washed with TBS-T, incubated with ECL reagents and signals were captured in the ChemiDoc system (Bio-Rad, Hercules, CA, USA). Membranes were subsequently stripped and incubated with anti-RyR2 antibodies as described above.

### Statistical Analysis

Values represent Mean ± SEM. Comparison between two groups was performed with Student’s *t*-test, and for multiple groups with one-way ANOVA followed by Tukey’s *post hoc* test or with two-way ANOVA followed by Bonferroni’s *post hoc* test; *p* < 0.05 was considered statistically significant.

## Results

**FIGURE 1:**
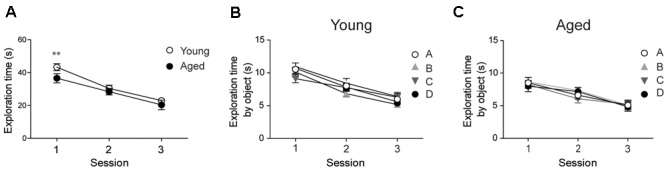
**Young and aged rats explored the four objects for similar periods and did not exhibit preference for any given object: (A)** At the second and third sessions, young (*n* = 44) and aged (*n* = 30) rats displayed similar average exploration times. Only at the first session the differences were statistically significant; ^∗∗^*p* < 0.01 [*F*_(1,144)_= 1.14]. **(B)** Young rats (*n* = 44) explored the four objects for equal times in each session. **(C)** Aged rats (*n* = 30) also explored the four objects for equal times in each session. In **(B,C)**, differences within sessions were not statistically significant. Values represent Mean ± SEM. Statistical analysis was done with two-way ANOVA followed by Bonferroni’s *post hoc* test.

Prior to assessing performance in the recognition tasks, we evaluated whether young and aged animals explored the behavioral apparatus and recognized the objects during the sample phase sessions. Both young and aged rats exhibited decreased exploration times in each of the three consecutive sample phase sessions tested; a comparison between both groups revealed significant differences only in the first session (**Figure 1A**). Young and aged rats showed similar exploration times for all four objects in the three sample phase sessions (**Figures 1B,C**).

### Aged Rats Have Impaired Long-Term Recognition Memory

**FIGURE 2:**
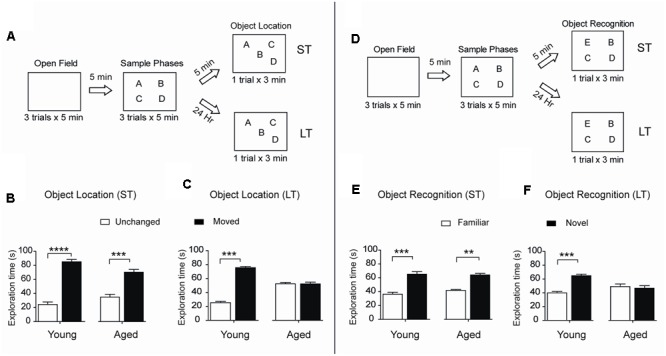
**Aged rats display impairments in long-term spatial memory and novel-object recognition: (A)** Diagram of the behavioral procedure used to test spatial memory retention in an object-location memory task. During the open field exploration phase, rats were habituated to the arena for 5 min during three consecutive days. In the sample phase, each rat was allowed to explore four different objects during 5 min for three consecutive sessions separated by 5 min intervals. Five minutes (ST: short-term assays) or 24 h (LT: long-term assays) after conclusion of the sample phase, independent groups of rats were exposed for 3 min to a new spatial arrangement, in which two of the four objects had different positions. **(B)** When tested after 5 min (ST), both young (*n* = 8), and aged (*n* = 7) rats discriminated the two spatially modified objects as evidenced by the increases in the time spent exploring the moved object. **(C)** When tested after 24 h (LT), young rats (*n* = 15) recognized the spatial rearrangement of objects and explored for longer times the moved object whereas aged rats (*n* = 8) did not. **(D)** Diagram of the behavioral procedures used to test novel-object recognition. The open field and the sample phases were as defined in **(A)**; for further details, see Section “Materials and Methods.” Five min or 24 h after the conclusion of the sample phase, independent groups of rats were exposed for 3 min to a different object arrangement, in which a new object replaced one of the four original objects. **(E)** When tested after 5 min (ST), both young (*n* = 8), and aged (*n* = 7) rats explored for longer times the novel-object compared with the three familiar objects. **(F)** When tested after 24 h (LT), young rats (*n* = 13) discriminated and explored for longer times the novel-object while aged rats (*n* = 8) did not. Values represent Mean ± SEM. Statistical significance was assessed by the Student’s *t*-test (^∗∗^*p* < 0.01; ^∗∗∗^*p* < 0.005; ^∗∗∗∗^*p* < 0.001).

To assess spatial memory retention in an object-location memory task, rats were tested 5 min or 24 h after concluding the sample phases, during which rats explored four different objects during 5 min for three consecutive sessions separated by 5 min intervals (**Figure 2A**). When tested after 5 min both young and aged rats recognized the two spatially modified objects and did not present significant differences in performance (**Figure 2B**). In contrast, only young rats recognized the spatial rearrangement of the objects when tested after 24 h (**Figure 2C**). We also evaluated possible changes over time in the ability to recognize novel objects, a PrhC-dependent task, and carried out short (5 min) and long-term (24 h) assays (**Figure 2D**). When tested at short-term, both young and aged rats recognized the novel object (**Figure 2E**). In contrast, 24 h after exposure to the sample phases, young rats discriminated the novel object but aged rats failed this task and presented significant differences in novel-object recognition compared to young rats (**Figure 2F**). Of the total 180 s allowed for the OL and the OR tasks, young and aged rats did not present significant differences between stationary times (**Table 2**). Altogether, these findings reveal that aged rats lose after 24 h the ability to perform both memory recognition tasks.

**Table 2:** Quiescent times displayed by young and aged rats exposed to the object location or the object recognition tasks.

Task	Young rats (s)	Aged rats (s)
Object location, short time	71 ± 3 (8)	75 ± 4 (7)
Object location, long time	84 ± 2 (15)	76 ± 2 (8)
Object recognition, short time	79 ± 3 (8)	74 ± 2 (7)
Object recognition, long time	76 ± 4 (13)	80 ± 3 (8)

*Values represent Mean ± SEM. The numbers of animals used in each case is given in parenthesis.*

### Performing a Long-term Object-Location Task Increased RyR2/3 mRNA Levels in the CA1 Hippocampal Region from Young Rats

**FIGURE 3:**
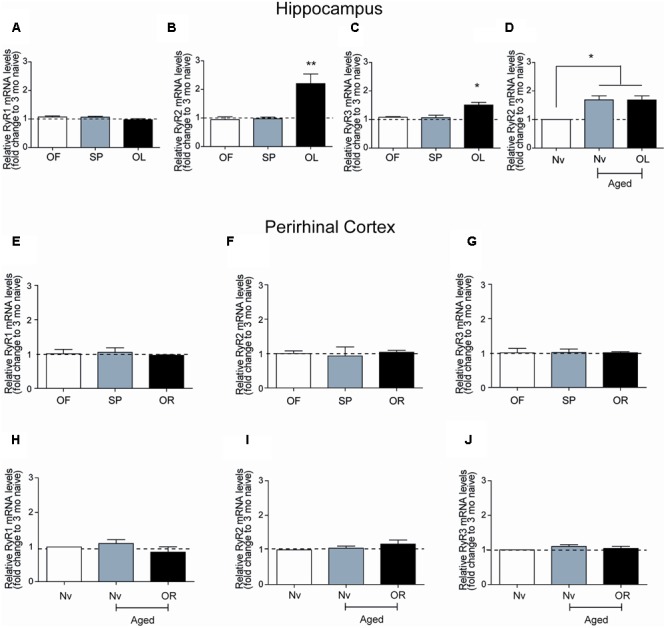
**Ryanodine receptor mRNA levels in young or aged rats exposed to recognition tasks: qRT-PCR analysis of extracts from the CA1 hippocampal region or the PrhC isolated from young or aged rats was performed 6-h after long-term exposure to the object-location (OL) or the novel-object recognition (OR) tasks.** All values for mRNA levels were normalized to the values displayed by naïve young rats. **(A)** CA1 RyR1 mRNA levels determined after the open field and the sample phases were similar to the values displayed by naïve young rats, and did not change after exposure to the OL task. **(B)** CA1 RyR2 mRNA levels determined after exposure of young rats to the open field and the sample phases were not statistically different to the values displayed by naïve young rats but increased after long-term exposure to the OL task. **(C)** CA1 RyR3 mRNA levels determined after exposure of young rats to the open field and the sample phases were similar to the values displayed by naïve young rats but increased after long-term exposure to the OL task. **(D)** In aged rats, RyR2 mRNA levels in the hippocampal CA1 region were higher than in naïve young rats and did not change after the OL task. In PrhC extracts from young or aged rats, RyR1 **(E,H)**, RyR2 **(F,I)** and RyR3 **(G,J)** mRNA levels determined after the open field and the sample phases were similar to the values displayed by naïve young rats; these values did not change after exposure to the OR task. OF, open field; SP, sample phases. Values represent Mean ± SEM; *n* = 3 for each group. Statistical significance was assessed by One-way ANOVA followed by Tukey’s *post hoc* test (^∗^*p* < 0.05; ^∗∗^*p* < 0.01).

The hippocampal CA1 region has a key role in learning and memory processes (Moser et al., 1995). After performing the long-term object-location task, young rats exhibited no changes in RyR1 mRNA levels in CA1 hippocampal micro dissections (**Figure 3A**), but presented significant increments in RyR2 mRNA levels (**Figure 3B**) and a more modest but significant increase in RyR3 mRNA levels (**Figure 3C**). Of note, the CA1 region from naive aged rats displayed significantly higher RyR2 (1.7-fold) mRNA levels relative to naïve young rats, which did not change after testing aged rats in the object-location task at long-term (**Figure 3D**). No changes in RyR1 and a modest increase (1.4-fold) in RyR3 mRNA levels in the CA1 region were also detected (data not shown); these levels did not change after long-term exposure to the object-location task.

### Novel-Object Recognition or Aging Do Not Modify RyR mRNA Levels in the Perirhinal Cortex

After performing at long-term the novel-object recognition task, which engages the PrhC, young rats did not exhibit significant differences in RyR1 (**Figure 3E**), RyR2 (**Figure 3F**), or RyR3 (**Figure 3G**) mRNA levels in the PrhC compared to naïve rats. In contrast to the increase in RyR2/RyR3 mRNA levels exhibited by the CA1 hippocampal region from naive aged rats, the PrhC from naïve aged rats had similar RyR1 (**Figure 3H**), RyR2 (**Figure 3I**), and RyR3 (**Figure 3J**) mRNA levels as naïve young rats. Moreover, these levels remained unaltered in the PrhC from aged rats tested after 24 h in the novel-object recognition task (**Figures 3H–J**).

### Performing the Object-Location Task or Aging Increase RyR2/RyR3 Protein Levels in the Hippocampus

**FIGURE 4:**
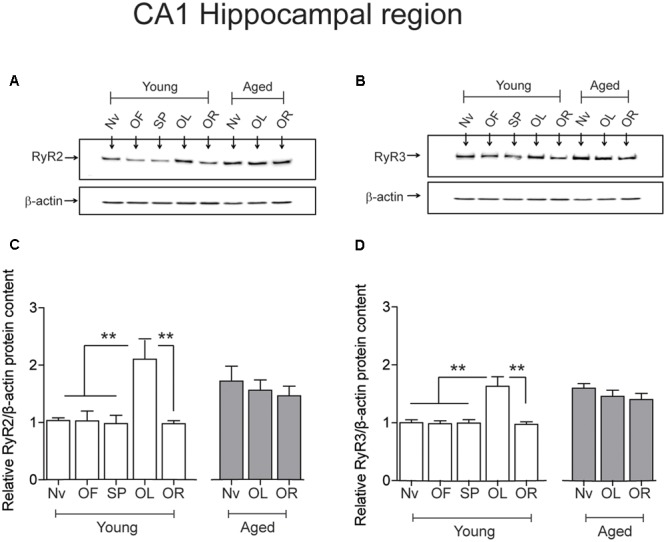
**Effects of performing the recognition tasks on hippocampal RyR2/RyR3 protein contents: Representative immunoblots of RyR2 (A)** and RyR3 **(B)** protein contents of extracts from the CA1 hippocampal region of young or aged rats; extracts were collected 6 h after long-term (24 h) exposure to the object-location (OL) or the novel-object recognition (OR) tasks. **(C)** In young rats, RyR2 protein content increased significantly after the OL [^∗∗^*p* < 0.01 (*n* = 7)] but not after the OR task (*p* > 0.05), and did not change in aged rats (*n* = 7) after exposure to either task (*p* > 0.05). **(D)** In young rats (*n* = 6), RyR3 protein content increased significantly in the CA1 region after the OL (^∗∗^*p* < 0.01) but not after the OR task (*p* > 0.05). Aged rats (*n* = 6) did not display changes in RyR3 protein content after performing either task (*p* > 0.05). OF, open field; SP, sample phases. Values represent Mean ± SEM. Statistical analysis was performed with one-way ANOVA followed by Tukey’s *post hoc* test.

After performing the long-term object-location task, the CA1 region from young rats exhibited significantly higher RyR2 (**Figures 4A,C**) and RyR3 (**Figures 4B,D**) protein contents. In contrast, neither the RyR2 (**Figures 4A,C**) nor the RyR3 (**Figures 4B,D**) protein levels changed after performing the novel-object recognition task. Naive aged rats exhibited significantly higher RyR2 (**Figures 4A,C**) and RyR3 (**Figures 4B,D**) protein contents in the CA1 region relative to young rats (**Table 3**); these levels did not change following exposure after 24 h to the object-location or the novel-object recognition tasks (**Figure 4**). We did not detect measurable RyR1 protein levels in the hippocampus from young or aged rats when using a highly specific antibody for the RyR1 isoform.

**Table 3:** Relative RyR2, RyR3, and IP_3_R1 protein contents.

	RyR2			RyR3			IP_3_R1		
	Young	Aged	*p*	Young	Aged	*p*	Young	Aged	*p*
CA1, Naive	1.03 ± 0.05 (7)	1.72 ± 0.26 (7)	<0.05	1.00 ± 0.05 (6)	1.59 ± 0.08 (6)	<0.0001	1.00 ± 0.08 (4)	1.14 ± 0.03 (4)	>0.05
CA1 Slice, Naïve	0.98 ± 0.03 (4)	1.42 ± 0.10 (4)	<0.005	0.96 ± 0.05 (4)	1.33 ± 0.10 (4)	<0.01	0.98 ± 0.33 (4)	0.98 ± 0.03 (4)	>0.05

*CA1 Slice: CA1 micro dissections from hippocampal slices. Numbers represent Mean ± SEM. In parenthesis, number of independent determinations, p values were generated by statistical comparison using unpaired One-tailed Student’s t-test.*

**FIGURE 5:**
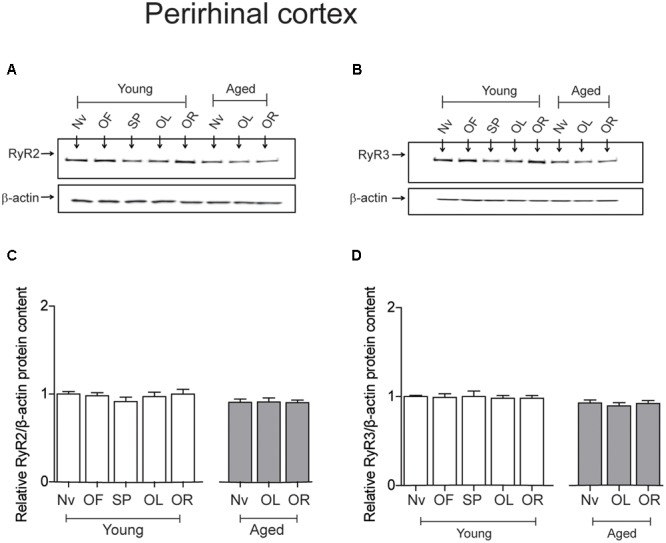
**Exposure to long-term memory tasks did not modify RyR2 and RyR3 protein contents in the PrhC from young or aged rats. (A,B)** Representative immunoblots of RyR2 and RyR3 protein contents in PrhC extracts from young or aged rats. Extracts were collected from naïve rats, or from rats exposed at long-term to the object-location (OL) or the novel-object recognition (OR) tasks. **(C)** Naïve (Nv) young (*n* = 7) or aged rats (*n* = 7) and trained young (*n* = 7) or aged rats (*n* = 7) displayed similar RyR2 protein contents. **(D)** Naïve (Nv) young (*n* = 6) or aged rats (*n* = 6) and trained young (*n* = 6) or aged rats (*n* = 6) displayed similar RyR3 protein contents. OF, open field; SP, sample phases. Values represent Mean ± SEM. Statistical analysis was performed with one-way ANOVA followed by Tukey’s *post hoc* test.

The PrhC isolated from young or aged rats had similar RyR2 protein contents. Likewise, both young and aged rats had similar RyR3 protein contents; these levels did not change after exposure to the object-location or the novel-object recognition tasks at long-term (**Figure 5**).

### Task- or Age-Related Changes in IP_3_R1 Protein Levels in the Hippocampus and the Perirhinal Cortex

**FIGURE 6:**
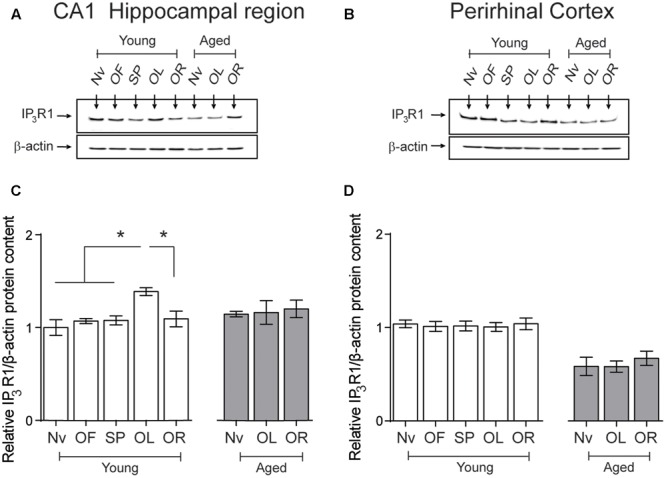
**Effects of performing the object location/novel-object recognition tasks on the IP_3_R1 protein content of the CA1 hippocampal region or the PrhC from young or aged rats: Representative immunoblots of the IP_3_R1 protein contents determined in (A)**, the CA1 hippocampal region or **(B)**, the PrhC isolated from young or aged rats. All samples were collected 6 h after exposure at long-term to the object-location (OL) or the novel-object recognition (OR) tasks. **(C)** The IP_3_R1 protein content of the CA1 hippocampal region from young rats increased after the OL (^∗^*p* < 0.05) but not after the OR task (*p* > 0.05); neither task modified the IP_3_R1 protein contents in the CA1 region from aged rats. **(D)** The IP_3_R1 protein content of the PrhC from young or aged rats did not change after exposure to the OL or the OR tasks (*p* > 0.05). OF, open field; SP, sample phases. Values represent Mean ± SEM (*n* = 4 in all groups). Statistical analysis was performed with one-way ANOVA followed by Tukey’s *post hoc* test.

After performing the object-location, but not the novel-object recognition task, young rats displayed a small but significant increment in IP_3_R1 protein levels in the hippocampal CA1 region, whereas aged rats did not present this task-related increase (**Figures 6A,C**). Naïve aged displayed similar IP_3_R1 protein contents as naïve young rats (**Table 3** and **Figures 6A,C**). The IP_3_R1 protein levels in young rats did not change in the PrhC after performing the object-location or the novel-object recognition tasks (**Figures 6B,D**). Interestingly, naïve aged rats displayed lower IP_3_R1 protein levels in the PrhC compared to young rats and these low levels did not change after exposure to the object-location or the novel-object recognition tasks at long-term (**Figures 6B,D**).

### Aged Rats Exhibit Altered Synaptic Transmission and Hippocampal Synaptic Plasticity

**FIGURE 7:**
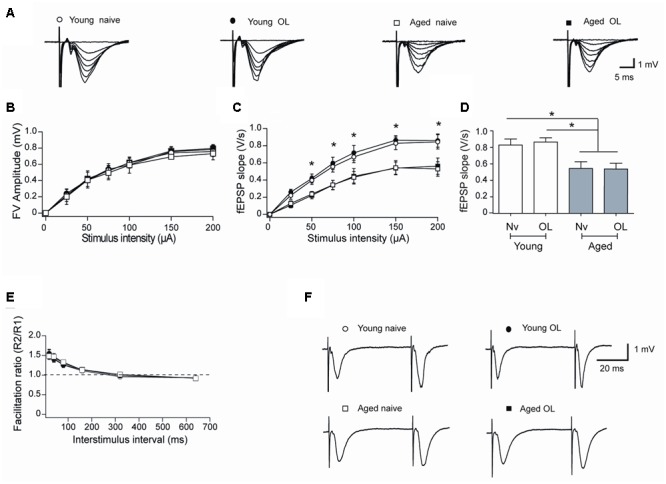
**Altered synaptic transmission (CA3-CA1) in aged rat hippocampus: (A)** Representative fEPSP traces elicited by different stimulus intensities recorded in rat hippocampal slices from young (open circles) or aged (open squares) naïve rats, or in slices isolated 6 h after long-term exposure of young (solid circles) or aged (solid squares) rats to the object-location (OL) task. **(B)** Relationship between stimulus intensity and FV amplitude recorded in slices from young or aged naive rats or in slices from young or aged rats after exposure to the OL task; symbols correspond to those defined in **(A)**. **(C)** fEPSP slopes versus stimulus intensity recorded in slices from young or aged naive rats, or in slices from young or aged rats collected after the OL task; symbols correspond to those defined in **(A)**. **(D)** Comparison of fEPSP slopes recorded when stimulating at 150 μA slices from young or aged naive rats, or recorded in slices from young or aged rats collected after the OL task (^∗^*p* < 0.05). **(E)** Paired-pulse facilitation responses recorded in the same four groups. Representative fEPSP traces registered at 40 ms inter-stimulus intervals; symbols correspond to those defined in **(A)**. **(F)** In these experiments, 23 slices from 7 young naive animals, 18 slices from 7 trained young rats, 16 slices from 6 aged naive animals and 14 slices from 6 aged trained rats were used. Values represent Mean ± SEM. Statistical analysis was performed by One-way ANOVA, followed by Tukey’s *post hoc* test.

Aging entails impairments in synaptic plasticity processes (Barnes, 2003; Rosenzweig and Barnes, 2003; Hidalgo and Arias-Cavieres, 2016). To inquire into the synaptic basis of these defects, we examined the strength and plasticity of CA3-CA1 synapses. To this purpose, we isolated rat hippocampal slices from naïve rats or 6 h after the conclusion of behavioral procedures and registered field fEPSP in the CA1 region (**Figure 7A**). Slices from aged and young rats showed similar increments in FV amplitude with increasing stimulus intensity (**Figure 7B**), but slices from aged rats displayed lower fEPSP slopes versus stimulus intensity when compared to slices from young rats (**Figure 7C**). Differences between fEPSP slopes measured in slices from young and aged rats at the stimulus intensity of 150 μA, a value that produced maximal responses, were statistically significant (**Figure 7D**), suggesting that aged rats present defects in basal synaptic transmission.

To test whether a decreased probability of neurotransmitter release contributes to the reduced basal transmission recorded in slices from aged rats, we evaluated the paired-pulse ratio, which reflects the quantal release of neurotransmitter from the presynaptic neurons (Schulz et al., 1994). Hippocampal slices from either young or aged rats did not exhibit differences in facilitation ratio (**Figures 7E,F**), suggesting that defective post-synaptic events underlie the reduction in basal synaptic transmission displayed by aged rats. In addition, hippocampal slices isolated from young or aged rats exposed at long-term to the object-location task did not exhibit differences in FV amplitude (**Figure 7B**), fEPSP responses (**Figure 7C**) or facilitation ratio (**Figures 7E,F**), when compared to their respective naïve counterparts.

**FIGURE 8:**
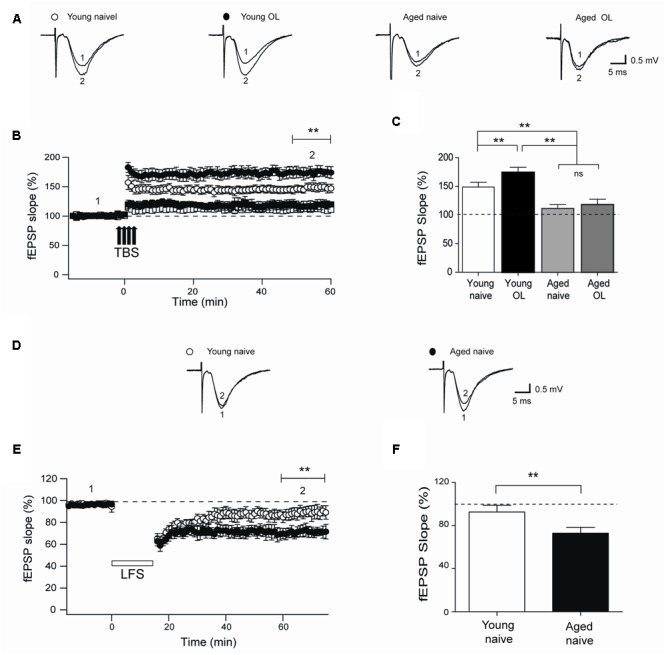
**Impaired LTP and enhanced LTD in slices from aged rat hippocampus: (A)** Representative fEPSP traces recorded 1–5 min before (trace 1) and 60 min after applying TBS (trace 2) in rat hippocampal slices from control young (open circles) or aged (open squares) rats. Six h after long-term exposure to the object-location (OL) task, fEPSP were recorded in slices isolated from young (solid circles) or aged (solid squares) rats. **(B)** Slices from trained young rats (solid circles; *n* = 7) showed significantly higher fEPSP slopes (^∗∗^*p* < 0.01) after 4-trains of TBS (arrows) when compared to slices from naive young rats (open circles; *n* = 7). Slices from naive (open squares; *n* = 6) or trained (solid squares; *n* = 6) aged rats displayed significantly lower fEPSP slopes (^∗∗^*p* < 0.01) than slices from young rats. **(C)** Average LTP magnitudes displayed during the last 10 min of recording (segment 2 in **B**) in slices from naive young (*n* = 23) or aged (*n* = 16) rats or from trained young (*n* = 18) or aged (*n* = 14) rats. **(D)** Representative fEPSP traces recorded in slices from young or aged rats 1–5 min (segment 1 in **E**) before LFS and 60 min after LFS (segment 2 in **E**). **(E)** Naïve aged rats displayed persistently lower fEPSP slopes respect to naive young rats after the LTD induction protocol (^∗∗^*p* < 0.01), delivered at the time indicated by the horizontal open bar. **(F)** Average magnitudes of fEPSP slopes recorded during the last 10 min in slices from young or aged naive rats, which displayed significantly lower values (^∗∗^*p* < 0.01). In **(E,F)**, 10 slices from 5 young animals and 12 slices from 6 aged rats were used. Values represent Mean ± SEM. Statistical significance of values in **(B,C)** was assessed with One-way ANOVA followed by Tukey’s *post hoc* test. Statistical analysis in **(E,F)** was performed with unpaired Student’s *t*-test.

Next, we evaluated TBS-induced LTP at the CA3-CA1 synapses in naïve rats and in rats exposed to the object-location task after 24 h. Compared to their naïve counterparts, trained young rats displayed significantly increased LTP (**Figures 8A,B**) and higher fEPSP responses measured 1 h after TBS (**Figure 8C**), suggesting that this particular behavioral protocol enhances synaptic plasticity as reported for other types of training protocols (Whitlock et al., 2006). In contrast, hippocampal slices from aged rat displayed impaired LTP (**Figures 8A,B**) and significantly lower fractional increase in fEPSP slopes regardless of previous exposure to the object-location task (**Figure 8C**).

In addition, we assessed LTD induction by LFS of slices from naïve young or aged rats. Slices from aged rats exhibited significantly increased LTD (**Figures 8D,E**), with significantly higher fractional decrease in fEPSP slopes (evaluated at the end of the record) in comparison to slices from young rats (**Figure 8F**).

### LTP and Aging – But Not LTD – Increase RyR2/RyR3 Protein Levels in the CA1 Region from Hippocampal Slices

**FIGURE 9:**
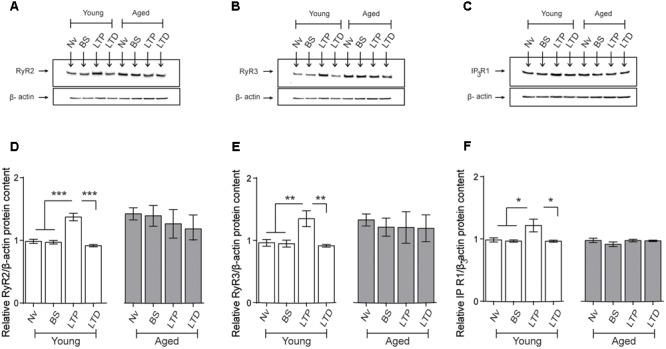
**Effects of LTP and LTD induction protocols on RyR2, RyR3, and IP_3_R1 protein contents in the CA1 hippocampal region: (A–C)** Representative immunoblots of RyR2, RyR3, and IP_3_R1 protein contents determined in extracts of the CA1 hippocampal region isolated from naïve young or aged rats. Extracts were collected 1 h after applying the LTP or LTD induction protocols. **(D)** In naïve young rats, the RyR2 protein content increased 1 h after LTP induction (^∗∗∗^*p* < 0.005) but did not change after LTD induction (*p* > 0.5); in aged rats, neither protocol modified the RyR2 protein content compared to the levels exhibited by naïve aged rats (*p* > 0.05). **(E)** In young rats, the RyR3 protein content increased 1 h after LTP induction (^∗∗^*p* < 0.01) but did not change after LTD induction (*p* > 0.05); in aged rats, neither protocol modified the RyR3 protein content (*p* > 0.05). **(F)** In young rats, the IP_3_R1 protein content increased 1 h after LTP induction (^∗^*p* < 0.05) but did not change after LTD induction (*p* > 0.05); in aged rats, these two protocols did not modify IP_3_R1 protein content (*p* > 0.05). Values represent Mean ± SEM (*n* = 4 animals for each condition). Statistical analysis was performed with one-way ANOVA followed by Tukey’s *post hoc* test.

We evaluated RyR2, RyR3, and IP_3_R1 protein contents in CA1 hippocampal micro dissections from slices isolated from young or aged rats that were exposed to LTP- or LTD-inducing protocols. After 1 h of exposure to the TBS protocol, slices from young rats displayed significant increases in RyR2 (**Figures 9A,D**), RyR3 (**Figures 9B,E**), and IP_3_R1 (**Figures 9C,F**) protein contents in the CA1 region. In contrast, basal stimulation or exposure to the LTD-inducing protocol did not modify the content of these three proteins in CA1 from young rat slices, measured after 1 h (**Figure 9**). As exhibited by the CA1 region isolated from the whole hippocampus of naive aged rats (**Table 3**), CA1 micro dissections from slices obtained from naïve aged rats also displayed increased RyR2 (**Figures 9A,D**) and RyR3 (**Figures 9B,E**) protein contents (**Table 3**). These levels did not change after basal stimulation or after exposure to either the LTP- or the LTD-inducing protocols.

In agreement with the results presented in **Table 3**, the IP_3_R1 protein content of the CA1 region from slices obtained from naive aged rats was not significantly different from that exhibited by naïve young rats (**Table 3** and **Figures 9C,F**). These levels did not change in response to basal stimulation or after exposure to the LTD-inducing protocol (**Figure 9**). In contrast, after LTP-inducing stimulation the IP_3_R1 protein content showed a modest but significant increase in CA1 dissections from hippocampal slices isolated from young but not from aged rats (**Figures 9C,F**).

### Aged Rats Display Higher RyR2 Oxidation Levels in the Hippocampus Compared to Young Rats

**FIGURE 10:**
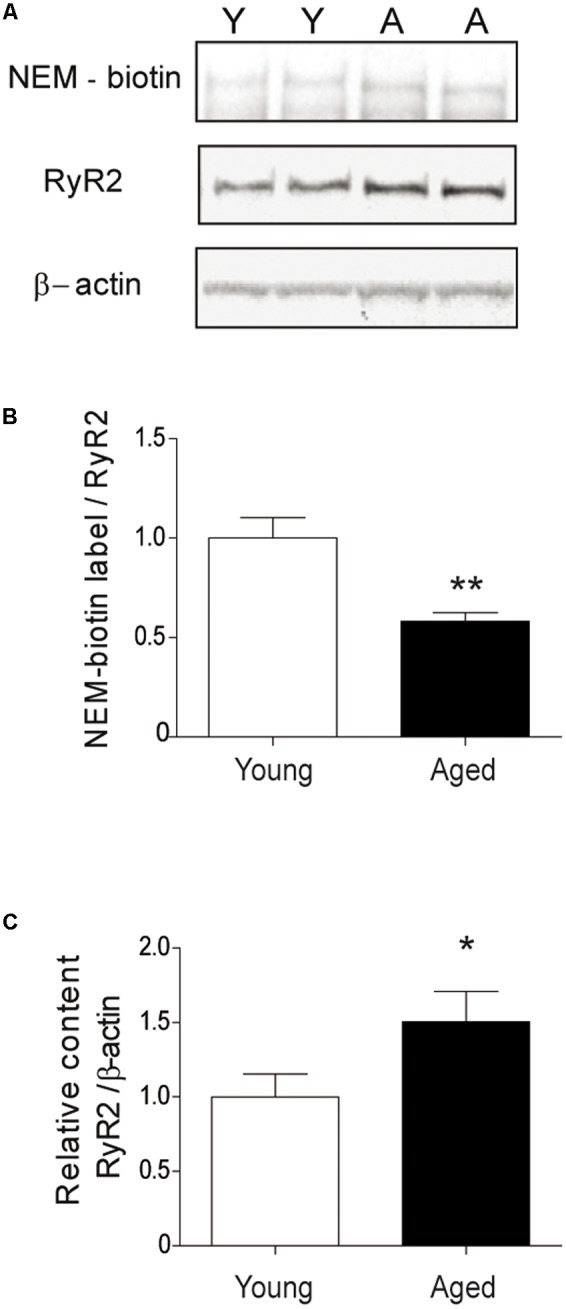
**The hippocampus from aged rats has RyR2 channels with higher oxidation levels. (A)** The figure illustrates a representative blot of duplicate hippocampal samples from young or aged rats, treated first with the streptavidin reagent for NEM-biotin labeling and then with antibodies against RyR2 and β-actin. **(B)** Quantification of band densities revealed by NEM-biotin labeling and antibodies against RyR2; values expressed as ratios were normalized to the values exhibited by young rats. **(C)** Quantification of band densities revealed with antibodies against RyR2 and β-actin; values represent Mean ± SEM of RyR2/β-actin ratios, normalized to the values exhibited by young rats. The whole hippocampus from either young (*n* = 6) or aged (*n* = 6) animals was analyzed for each condition. Statistical analysis of results presented in **(B,C)** was performed with unpaired One-tailed Student’s *t*-test. ^∗^*p* < 0.05; ^∗∗^*p* < 0.01.

We determined if the RyR2 channels present in aged rat hippocampus displayed increased oxidation levels, an unexplored subject to our knowledge. We found that the RyR2 channels expressed in the hippocampus isolated from aged rats exhibited significantly higher oxidation levels relative to the levels displayed by RyR2 channels present in young rat hippocampus (**Figure 10**).

## Discussion

The novel findings reported in this work are the selective hippocampal increases in RyR2/RyR3 and IP_3_R1 protein contents induced by performance of young rats in a hippocampus-dependent spatial memory task, and following LTP but not LTD induction. We show, in addition, that the CA1 hippocampal region from aged rats has increased RyR2/RyR3 protein contents and that these levels do not change after testing aged rats at long-term in the spatial memory task or following LTP or LTD induction. Moreover, RyR2 oxidation levels in the hippocampus from aged rats are higher than in young rat hippocampus. We also show that the PrhC from young rats expresses RyR2, RyR3, and IP_3_R1 channels and that these levels are not modified by age, or by exposing at 24 h young or aged rats in the object-location or the novel-object recognition tasks. Additionally, aged rats successfully performed the object-location and the novel-object recognition tasks at short-term (5 min) but failed both tasks when tested after 24 h.

### Aged Rats Display Cognitive Impairments and Changes in Hippocampal Synaptic Plasticity

Previous reports have shown that hippocampal-dependent spatial memory deteriorates during aging (Rosenzweig and Barnes, 2003; Veng et al., 2003; Wimmer et al., 2012; Kumar and Foster, 2013; Hopp et al., 2014). In agreement with these reports, we found that aged rats presented impaired hippocampal-dependent spatial long-term memory. In contrast to the general agreement on the deleterious effects of aging on hippocampal-dependent spatial memory, studies on the impact of aging on PrhC-dependent object recognition memory have yielded contradictory information. Previous reports using short delay intervals between the sample phase session and the object recognition task indicate that aged rats effectively discriminate novel objects (Cavoy and Delacour, 1993; Lukaszewska and Radulska, 1994). In contrast, studies using longer delay intervals report that aged rats fail this task (Burke et al., 2010; Gamiz and Gallo, 2012). Our results contribute to solve this apparent contradiction, since we found that the interval duration between the sample phase session and the object recognition task determines the response of aged rats. Thus, when examined after 5 min aged rats discriminated the novel object but failed to do so when evaluated after 24 h.

Synaptic plasticity is likely to represent the neuronal substrate for learning and memory (Gruart et al., 2006; Whitlock et al., 2006). Aging causes a progressive decline in synaptic function and causes significant LTP impairments, which correlate with the impaired ability to process and store information (Robillard et al., 2011; Haxaire et al., 2012). Here, we report reductions in synaptic strength and faulty LTP in aged animals; these responses strongly correlate with their spatial cognitive dysfunction. These deleterious changes probably arise from defects in post-synaptic mechanism, since presynaptic-dependent properties, such as the amplitude of FVs and the paired pulse responses were similar in young and aged rats. We also confirmed that aged rodents display increased susceptibility to LTD induction, as previously reported (Norris et al., 1996; Kumar and Foster, 2005).

### Training in Spatial Memory Tasks or Sustained LTP Induction Increases Hippocampal RyR2, RyR3, and IP_3_R1 Protein Contents Only in Young Rats

A significant increase in hippocampal RyR2 (Zhao et al., 2000; Adasme et al., 2011) and RyR3 (Adasme et al., 2011) mRNA and protein levels occurs in young rats trained in the Morris water maze, a widely accepted protocol to evaluate hippocampal-dependent spatial memory (Morris et al., 1982). In addition, increased levels of RyR1, RyR2, and RyR3 mRNA correlate negatively with the performance of aged rats in this maze (Hopp et al., 2014). Our results expand these previous findings by showing that successful performance of a different hippocampal-dependent spatial memory task promoted significant increases in the mRNA and protein levels of RyR2/RyR3 in young rat hippocampus. The IP_3_R1protein content also increased in the hippocampal CA1 region from young rats after successful performance at long-term of the spatial memory task. Up-regulation of these Ca^2+^ release channels caused by the spatial memory task was restricted to the hippocampus, since their levels did not change in the PrhC isolated from the same groups of trained young rats. Moreover, successful performance at long-term of the PrhC-dependent novel-object recognition task did not modify the expression of these channels in the PrhC from young rats. Consequently, we propose that successful performance of the spatial memory task selectively engages hippocampal cellular pathways that promote RyR2/RyR3 and IP_3_R1 up-regulation in young rats. In addition, sustained LTP (1 h) but not LTD resulted in increased RyR2, RyR3, and IP_3_R1 protein contents in hippocampal slices from young rats. To our knowledge, this is the first description of hippocampal up-regulation of these two types of calcium release channels induced both by LTP and by a spatial memory task.

### Aged Rats Display Increased RyR2/RyR3 Protein Content and Higher RyR2 Oxidation Levels

The expression of several genes changes during the aging process (Kadish et al., 2009). In particular, aging entails decreased levels of FK506-Binding Protein 12.6 (FKB12.6), a RyR-associated protein that inhibits RyR channel activity; this decrease correlates with cognitive decline while FKBP12.6 overexpression reverses it (Gant et al., 2015). Synaptic dysfunction correlates with altered Ca^2+^ signaling in aged rodents, affecting in turn neuronal excitability and learning (Disterhoft et al., 1996; Thibault and Landfield, 1996; Foster and Norris, 1997; Gant et al., 2015). Enhanced RyR-mediated CICR (Kumar and Foster, 2005; Gant et al., 2006) and increased L-type Ca^2+^ channel expression (Veng et al., 2003) and activity (Thibault and Landfield, 1996) contribute to age-related Ca^2+^ signaling dysregulation. Our results reveal increased RyR2/RyR3 protein contents in the hippocampus but not in the PrhC from naive aged rats. In contrast, aged rats had similar hippocampal IP_3_R1 protein content but decreased levels of this protein in the PrhC relative to young rats.

In addition to defective Ca^2+^ signaling, enhanced ROS generation is a characteristic trait of the aging process, which if uncontrolled leads to neuronal oxidative stress. Age-related changes in synaptic plasticity correlate with oxidative stress and with post-synaptic shifts toward oxidation in the intracellular oxidation-reduction environment (Bodhinathan et al., 2010a,b; Robillard et al., 2011; Haxaire et al., 2012; Kumar and Foster, 2013). In particular, the increased oxidative tone present in aged neurons decreases the synaptic responses induced by activation of N-methyl-D-aspartate receptors and affects as well other mechanisms underlying synaptic plasticity (Bodhinathan et al., 2010a; Kumar and Foster, 2013). We show here that RyR2/RyR3 protein content and RyR2 oxidation levels are higher in the hippocampus from aged naïve rats compared to naive young rats. Of note, RyR oxidative modifications strongly stimulate RyR-mediated Ca^2+^ release whereas reducing agents have the opposite effects (Hidalgo and Donoso, 2008; Hidalgo and Arias-Cavieres, 2016). Accordingly, we propose that the increase in RyR2/RyR3 hippocampal protein content and in RyR2 oxidation displayed by aged neurons, plus their decreased FKB12.6 levels (Gant et al., 2015), jointly contribute to produce the enhanced RyR-mediated Ca^2+^ signals that cause the prolonged sAHP phase exhibited by aged CA1 pyramidal neurons (Bodhinathan et al., 2010b). Since reducing agents significantly ameliorate the RyR activity-dependent prolonged sAHP phase exhibited by aged neurons (Bodhinathan et al., 2010b), we suggest that restoring RyR-mediated Ca^2+^ release to normal levels would reverse, at least partially, the deleterious effects of aging on hippocampal neuronal function.

## Conclusion

Based on the present findings, we propose that successful spatial memory acquisition and sustained LTP induction require the increased expression of RyR2/RyR3, and possibly of IP_3_R1 Ca^2+^ release channels as well. A direct test of this hypothesis would require selective inhibition of the increase in the expression of these channels induced by neuronal activity without affecting their basal levels, which have key roles in hippocampal LTP and spatial memory processes (Paula-Lima et al., 2014). Strategies to inhibit selectively activity-induced RyR2 or RyR3 up-regulation are not readily apparent. The transcription factor cyclic AMP response element binding protein (CREB) drives nicotine-mediated selective RyR2 up-regulation in brain areas associated with cognition and addiction (Ziviani et al., 2011). However, inhibition of activity-related RyR2 up-regulation via CREB inhibition would lack in selectivity, because CREB mediates the expression of many neuronal genes. In addition, information regarding the factors that control RyR3 expression is not available. Nevertheless, the current findings showing that aged rats exhibited impaired LTP and spatial memory and did not present the accompanying up-regulation of Ca^2+^ release channels support the proposal that hippocampal RyR up-regulation is essential for sustained LTP and spatial memory processes. In addition, we propose that the increased RyR2/RyR3 hippocampal expression and the higher RyR2 oxidation levels displayed by untrained aged rats contribute to generate the anomalous Ca^2+^ signals reported in neurons from aged hippocampus (Gant et al., 2006; Bodhinathan et al., 2010b). These anomalous Ca^2+^ signals presumably prevent the activity-induced changes in RyR2/RyR3 expression that occur in young rats, among other harmful effects, and thus are likely to contribute to the spatial memory and LTP defects exhibited by aged rats.

## Author Contributions

CH supervised and funded this work, designed experiments, analyzed the experimental results, and wrote the manuscript. AA-C designed and carried out most of the experiments, analyzed the experimental results, and contributed to manuscript writing. TA performed and analyzed all qPCR experiments, and contributed to manuscript writing. GS performed the analysis of RyR channel redox state, analyzed results and contributed to manuscript writing. PM supervised and designed experiments, analyzed the experimental results, and contributed to manuscript writing.

## Conflict of Interest Statement

The authors declare that the research was conducted in the absence of any commercial or financial relationships that could be construed as a potential conflict of interest.

## Abbreviations

CICR: Ca^2+^-induced Ca^2+^ release
fEPSP: field excitatory post-synaptic potentials
FV: fiber volley
IP_3_R1: inositol 1,4,5-trisphosphate receptor type-1
LFS: low frequency stimulation
LTD: long-term depression
LTP: long-term potentiation
PrhC: perirhinal cortex
ROS: reactive oxygen species
RyR2/RyR3: ryanodine receptor types-2/3
sAHP: slow after-hyperpolarization
TBS: theta burst stimulation.
